# DAL-1 attenuates epithelial-to mesenchymal transition in lung cancer

**DOI:** 10.1186/s13046-014-0117-2

**Published:** 2015-01-22

**Authors:** Xianliang Chen, Xiaoying Guan, Huiyu Zhang, Xiaobin Xie, Hongyan Wang, Jie Long, Tonghui Cai, Shuhua Li, Zhen Liu, Yajie Zhang

**Affiliations:** Department of Pathology, School of Basic Medical Science, Guangzhou Medical University, 195# Dongfeng West Road, Guangzhou, Guangdong 510180 People’s Republic of China

**Keywords:** Differentially expressed in adenocarcinoma of the lung-1, Epithelial-mesenchymal transition, Lung cancer, E-cadherin, HSPA5

## Abstract

**Background:**

Epithelial-to mesenchymal transition (EMT) involves in metastasis, causing loss of epithelial polarity. Metastasis is the major cause of carcinoma-induced death, but mechanisms are poorly understood. Here we identify differentially expressed in adenocarcinoma of the lung-1 (DAL-1), a protein belongs to the membrane-associated cytoskeleton protein 4.1 family, as an efficient suppressor of EMT in lung cancer.

**Methods:**

The relationship between DAL-1 and EMT markers were analyzed by using immunohistochemistry in the clinical lung cancer tissues. Quantitative real-time PCR and western blot were used to characterize the expression of the EMT indicator mRNAs and proteins in DAL-1 overexpressed or knockdown cells. DAL-1 combined proteins were assessed by co-immunoprecipitation.

**Results:**

DAL-1 levels were strongly reduced even lost in lymph node metastasis and advanced pathological stage of human lung carcinomas. Overexpression of DAL-1 altered the expression of numerous EMT markers, such as E-cadherin, β-catenin Vimentin and N-cadherin expression, meanwhile changed the morphological shape of lung cancer cells, and whereas silencing DAL-1 had an opposite effect. DAL-1 directly combined with E-cadherin promoter and regulated its expression that could be the reason for impairing EMT and decreasing cell migration and invasion. Strikingly, HSPA5 was found as DAL-1 direct binding protein.

**Conclusions:**

These results suggest that tumor suppressor DAL-1 could also attenuate EMT and be important for tumor metastasis in the early transformation process in lung cancer.

**Electronic supplementary material:**

The online version of this article (doi:10.1186/s13046-014-0117-2) contains supplementary material, which is available to authorized users.

## Background

Lung cancer is the leading cause of cancer-related mortality worldwide [1]. The main cause of death is the invasion and metastasis of tumor. EMT is the early event of lung cancer metastasis which is characterized by a loss of cell-cell adhesion and polarity, down-regulation of epithelial markers, and acquisition of mesenchymal markers and phenotype [2]. Thus, a better understanding of cellular and molecular mechanisms leading to metastatic dissemination of carcinoma cells is of utmost importance.

The human tumor suppressor gene DAL-1 was identified using Differential Display PCR as a gene whose expression was lacking in non-small cell lung cancer when compared with matched normal tissue [3]. Frequent loss of DAL-1 in cervical cancer [4], laryngeal squamous cell carcinoma [5], breast cancer [6] and esophageal squamous cell carcinoma [7] suggested that DAL-1 could be a tumor suppressor [8,9]. It has also been reported that loss of DAL-1 expression and methylation of the DAL-1 promoter are involved in development and progression of NSCLC (Non small cell carcinoma), providing a possible indicator of poor prognosis. Further studies demonstrated DAL-1 contained FERM (protein 4.1-ezrin-radixin-moesin) and SABD (spectrin-actin-binding domain) domain [10], enable it links to the transmembrane protein and cytoskeleton. Consequently, DAL-1 was considered as membrane associated protein contributing to cell shape, cell-cell and cell-matrix adhesion, and cell motility. DAL-1 also participates in the organization of cytoskeleton [6,11]. E-cadherin the key role in EMT and DAL-1 co-locate in the junction part of cells, therefore, loss of DAL-1 may associate with disruption of cell-cell adhesion, loss of polarity and cancer metastasis.

Herein, the effect of DAL-1 on EMT was identified, and its role on regulating EMT was illuminated in lung cancer cell lines. In this paper, we showed that expression of DAL-1 was downregulated in metastatic tumours and further identified DAL-1 as an EMT/metastasis suppressor.

## Material and methods

### Tissue samples and cell lines

The paraffin-embedded tissues of 190 primary lung cancers and 163 matched corresponding tumor adjacent tissues were obtained from patients who underwent primary surgical treatment without systemic chemotherapy or radiotherapy during the period 2005–2008. This project had the informed consents from all the patients and was approved by the First Affiliated Hospital of Guangzhou Medical University and informed consent was taken from all subjects. The average age of the patients at diagnosis was 60.34 years, ranging from 25 to 82 years. These cases included 77 squamous cell carcinomas (36 cases were well or moderately differentiated, 41 cases were poor differentiated), 85 adenocarcinomas (59 cases were well or moderately differentiated, 26 cases were poor differentiated), 11 adeno-squamous carcinomas (moderately differentiated), 6 small cell carcinomas (poor differentiated), 5 large-cell carcinomas (poor differentiated), 6 sarcomatoid carcinomas (poor differentiated). All samples were diagnosed and classified according to the World Health Organization grading system and the General Rules for Clinical.

The cell lines used in this study were purchased from American Type Culture Collection (ATCC).

### Plasmids and stable cell lines

Plasmid pcDNA3-DAL-1 containing full-length DAL-1 coding region was kindly provided by Dr. Philip Washbourne (University of Oregon, USA). This plasmid was verified by DNA sequencing. The control vector pcDNA3 was purchased from Invitrogen (Grand Island, NY, USA). Lipofectamine® LTX & Plus Reagent (Invitrogen, Carlsbad, CA, USA) was used to transfect. Transfected condition was pre-optimized using pEGFP-C1 (Clontec, Mountain View, CA, USA) to monitor the transfection efficiency under inverted fluorescent microscopy.

To obtain stable transfectants, cells seeded in six-well plates were transfected with 2.5 μg/well plasmids using 10 μl/well Lipofectamine®LTX Reagent and 2.5 μl/well Plus Reagent. After 6 h incubation in serum and antibiotic free condition, the medium was replaced with RPMI 1640 containing 10% FBS, and the cells were cultured for 48 h before submitting to a 2-week selection in medium containing G418 (600 μg/ml).

### Immunohistochemical staining

5-μm sections of paraffin-embedded tissues were treated with pepsin or 30 min and blocked with BSA for 30 min at room temperature. Tissue sections were then incubated with antibodies to DAL-1, E-cadherin, Snail or vimentin overnight at 4°C. Isotype control antibody (Sigma-Aldrich) was used as a negative control. Each slide was washed three times in TBS and incubated with biotinylated anti–mouse IgG (Vector Laboratories) in a humid chamber for 30 min. The positive cells were detected using peroxidase-conjugated streptavidin (Vector Laboratories) followed by 3′3-diaminobenzidine tetrahydrochloride (DAB; Vector Laboratories). The slides were counterstained with hematoxylin.

### Dual-luciferase reporter assay

The E-cadherin promoter (643 bp, from-450 to +193) was cloned into pGL3-Enhancer vector (Promega). A549 cells were plated in 24-well plates at a density of 1 × 10^5^ cells/well and grown overnight prior to transfection. The cells were transfected with either DAL-1 plasmid or control plasmid, and were co-transfected with pGL3-E-cadherin promoter plasmid and pGL3-TK plasmid. Forty-eight hours after transfection, the luciferase activities were analyzed by the Dual-luciferase system according to the manufacturer’s instruction (Promega). Three independent experiments were performed.

### Chromatin immunoprecipitation analysis

ChIP analysis was carried out as described previously [12]. Briefly, Protein-DNA complexes were immunoprecipatited with DAL-1 antibody, with human IgG as a control, respectively. ChIP DNA was isolated with the Qiaquick PCR Purification Kit (Qiagen). Subsequent qPCR using 1⁄50 fraction of ChIP-enriched DNA and 100 nM primers in a total volume of 20 μl was conducted to assess the amount of DNA that had been precipitated. Standard curves from 0.1-100 ng of sonicated genomic DNA were also amplified by qPCR as a reference. DNA from these samples was then subjected to PCR analysis. Primers sets for E-cadherin promoter were 5′-gca gac tgg aag ggc gg-3′ and 5′-ggc agg cag ccc agc-3′.

### Co-immunoprecipitation and mass spectrometric assay and protein identification

Co-immunoprecipitation was performed using Pierce® Co-IP kit (Thermo scientific, USA) following the manufacturer’s instruction. A549 cell lines stable tranfected either DAL-1 or control were lysed in ice-cold IP Lysis/Wash Buffer. After centrifugation at 13,000 g at 4°C for 10 min, the supernatant was collected. Pre-clear lysate using the control agarose resin before co-immunoprecipitation by the resin coupled with goat anti-DAL-1 polyclonal antibody. The immunoprecipitate was isolated by the elution buffer and separated by 10% SDS-PAGE. Sliver staining was used to detect the protein on the gel. The protein bands were submitted for tryptic peptide mass fingerprinting for identification by matrix-assisted laser desorption/ionization time-of-flight/time of flight mass spectrometry (MALDI-TOF/TOF-MS). Protein identification was performed by searching protein databases of Mascot (http://www.matrixscience.com).

Cells were lysed on ice for 30 min with a lysis buffer (150 Mm NaCl, 25 mM Hepes, pH 7.4, 10 mM NaF, 5 mM MgCl_2_ 1 mM EGTA, 1% Nonidet P-40, protease inhibitors mixture:10 μg/ml aprotinin, 10 μg/ml leupeptin, 1 mM PMSF). Total lysates were precleared with protein G-Sepharose for 1 h at 4°C and then immunoprecipitated overnight with 2 μg of anti-Myc antibody (Santa Cruz Biotechnology). Protein G-Sepharose was then added, and the mixture was incubated for an additional 1 h at 4°C. The beads were washed three times with lysis buffer, followed by elution in Laemmli buffer. The proteins were subjected to SDS-PAGE and Western blotting according to standard procedures.

### Migration and invasion assay

Cells were transfected with DAL-1, DAL-1 RNAi or negative control using Transfection Agent (Ambion, TX, USA) following manufacture’s protocol in 24-well plates. 24 h after transfection, Transwell migration assay and Matrigel invasion assay were performed separately using 24-well Transwell inserts with 8 μm pore size (Corning Costar Corp). For Transwell migration assay, 2 × 10^4^ A549 or H460 cells suspended in 100 μl corresponding culture medium without fetal bovine serum (FBS) were loaded into the top chamber of transwell insert with non-coated membrane. For Matrigel invasion assay, 5 × 10^4^ A549 or H460 cells were plated in 100 μl serum-free medium in the upper Matrigel-coated chamber instead. In both assays, the bottom chamber was containing 600 μl medium with 20% FBS. Cells were then allowed to migrated or invaded for 12 h at 37°C. The cells that migrated or invaded into the bottom chamber were fixed, stained with 1% nuclear fast red staining solution, visualized under phase contrast microscope and photographed. Total number of migrated or invaded cells was counted by IPP (Image-Pro Plus 6.0) software. All experiments were independently repeated at least three times.

### Statistical analysis

Data were expressed as the mean ± SD for each group. The difference between groups was analyzed using a factorial model one-way analysis of variance. SPSS statistical software, version19.0 (Chicago, Illinois, USA) was used to carry out all analyses and *P* less than 0.05 was considered statistically significant. Each experiment was performed in triplicate.

## Results

### DAL-1 is down-regulated in lung cancer tissues

**Figure 1:**
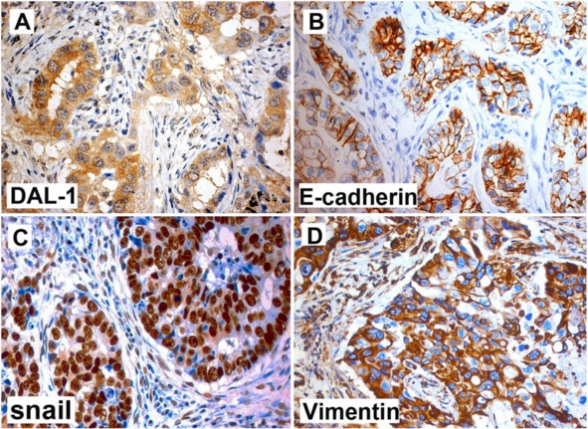
**Immunohistochemical staining on paraffin-embedded lung cancer tissue array sections.** Tissue sections of lung cancer were analyzed by IHC staining. The positive staining was shown in brown color. The sections were counterstained with hemotoxylin to show nuclei. Representative images of DAL-1 staining is shown that lower expression of DAL-1 **(A)** and E-cadherin **(B)** could been seen in well- differentiated adenocarcinoma, while positive expressions of snail **(C)** and Vimentin **(D)** were mainly localized in cytoplasm or nucleus of the serous lung cancer cells. Magnification, ×200.

Several kinds of lung cancer cell lines, including A549, SPC-A1, PGC-L3, GLC-82, H1299, L78, and NCI-H460 were analyzed the DAL-1 mRNA and protein expression levels. GLC-82 and NCI-H460 cell lines were positive for DAL-1 mRNA and protein expression as indicated by RT-PCR products at the expected size of 153 bp and the specific protein binding band at the predicted size of 108 kDa. The endogenous DAL-1 expression had not been found in other kinds of cell lines (Additional file 1). To investigate the clinical importance of DAL-1 expression in lung cancer tissues and to explore its role on EMT, the expression of E-cadherin, Vimentin and Snail were examined on 190 cases of lung cancer tissues and 163 adjacent tissues samples by immunohistochemistry analysis (Figure 1). Lower expression of DAL-1 was found in tumor tissues compared with the normal adjacent tissues; higher expression of DAL-1 was found in well differentiated lung cancer tissues compared with poorly differentiated lung cancer tissues. Data present from Table 1 showed that expression of E-cadherin was lower (*P* < 0.05), whereas Vimentin and Snail protein expression levels (*P* < 0.05) were higher in poorly differentiated lung cancer tissues. Meanwhile, positive staining of DAL-1 protein was preferentially observed in tumors without lymph node metastasis (81/98, 82.6%) relative to those with lymph node metastasis (52/92, 56.5%, *P* < 0.05). Positive DAL-1 staining was significantly higher in tumors with pathological stages I (69/83, 83.1%) than in those with stage II, III and IV (77 of 107, 72.0%, *P* < 0.05).

**Table 1:** **Correlation of DAL-1, E-cadherin, vimentin, snail and clinical pathological characteristics**

**Clinical pathological characteristics**	**n**	**DAL-1**					**E-cadherin**					**Vimentin**					**Snail**				
		**+++**	**++**	**+**	**-**	***P***	**+++**	**++**	**+**	**-**	***P***	**+++**	**++**	**+**	**-**	***P***	**+++**	**++**	**+**	**-**	***P***
Diameter (cm)																					
>3	109	16	33	23	37	0.426	13	16	13	67	0.464	9	19	18	63	*0.030*	50	29	16	14	*0.009*
≤3	81	10	32	19	20		7	10	16	48		2	6	11	62		21	24	12	24	
Differentiation																					
Well/mild differentiated	106	18	50	19	19	*0.000*	16	17	21	52	*0.003*	0	5	15	86	*0.000*	36	27	17	26	0.263
Poor differentiated	84	8	15	23	38		4	9	8	63		11	20	14	39		35	26	11	12	
Lymph metastasis																					
N0	98	22	43	16	17	*0.000*	12	17	24	45	*0.000*	0	10	6	82	*0.000*	24	30	15	29	*0.000*
N1	92	4	22	26	40		8	9	5	70		11	15	23	43		47	23	13	9	
Pathological stage																					
I	83	18	39	12	14	*0.000*	9	14	22	38	*0.015*	0	8	5	70	*0.000*	17	25	13	28	*0.000*
II	57	3	17	18	19		6	6	5	40		4	5	12	36		21	16	13	7	
III	42	5	8	9	20		5	5	1	31		5	11	11	15		28	9	2	3	
IV	8	0	1	3	4		0	1	1	6		2	1	1	4		5	3	0	0	

Spearman rank correlation analysis was applied to analyze the expression levels of DAL-1, E-cadherin, Snail, vimentin in lung cancer tissues (Table 2). The expression of DAL-1 was positively correlated with E-cadherin (r =0.710, *P* < 0.01), while negatively correlated with Vimentin (r = −0.499, *P* < 0.01) and Snail (r = −0.318, *P* < 0.01).

**Table 2:** **Correlation of DAL-1, E-cadherin, vimentin, snail and clinical histology types**

**Group**	**n**	**DAL-1**					**E-cadherin**					**Vimentin**					**Snail**				
		**+++**	**++**	**+**	**-**	***P***	**+++**	**++**	**+**	**-**	***P***	**+++**	**++**	**+**	**-**	***P***	**+++**	**++**	**+**	**-**	***P***
**Tissue distribution**																					
Lung cancer	190	26	65	42	57	*0.000*	20	26	29	115	*0.000*	11	25	29	125	*0.000*	71	53	28	38	*0.000*
Para-carcinoma tissue	163	86	59	4	14		81	54	8	20		0	0	0	163		28	25	14	96	
**Histology types**																					
Squamous cell carcinoma	77	11	30	17	19	0.168	9	12	10	46	0.534	3	9	12	53	0.422	3	9	12	53	0.301
Small cell carcinoma	6	0	0	1	5		0	1	0	5		0	1	0	5		0	1	0	5	
Adenocarcinoma	85	14	35	16	20		11	12	16	46		2	8	13	62		2	8	13	62	
Large cell carcinoma	5	1	0	1	3		0	1	0	4		2	1	0	2		2	1	0	2	
Adenosquamous carcinoma	11	0	0	5	6		0	0	2	9		3	2	4	2		3	2	4	2	
Carcinoma sareomatodes	6	0	0	2	4		0	0	1	5		1	4	0	1		3	1	1	1	

Based on our findings, the expression of DAL-1 is downregulated in lung cancer compared to normal tissues and significantly associated with disease progression, we suggest that decreased expression of DAL-1 may occur in the early stages of tumor development as well as be essential in maintaining tumorigenesis.

### DAL-1 overexpression alters the expression of EMT markers

**Figure 2:**
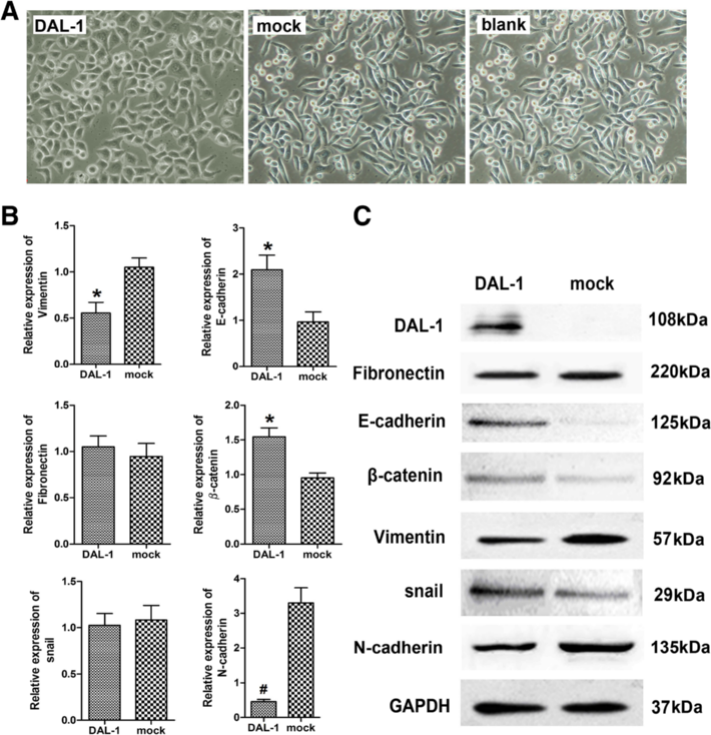
**Effect of DAL-1 overexpression on morphology and EMT markers in A549 cells.** Cells were stably transfected with pcDNA3-DAL-1 or pcDNA3 plasmid respectively. **A**, Morphological changes in DAL-1 overexpression cells, from spindle-like mesenchymal morphology into epithelial morphology, by manifesting an increased cell-to-cell adhesion; **B**, Q-PCR analysis of the levels of EMT related markers after DAL-1stably transfected with pcDNA3-DAL-1 plasmid; The levels of E-cadherin and β-catenin mRNAs were higher in the cells treated with the pcDNA3-DAL-1 plasmid than in the pcDNA3 treated cells (mock) or A549 cells (blank), but the level of Vimentin and N-cadherin mRNAs were lower. The data are presented as the mean ± SD. n = 3, *P <0.05,#*P* <0.01. **C**, Western blot analysis of DAL-1 and EMT marker protein expression levels in A549 cells. When the cells were transfected with the pcDNA3-DAL-1, the protein levels of E-cadherin and β-catenin increased while protein levels of Vimentin N-cadherin were down-regulated.

To verify whether DAL-1 is a crucial mediator of lung cancer, we established A549 cell line stably overexpressing DAL-1. We then analyzed DAL-1/A549 cells and the control cells through cell morphological observation. In the initial characterization of these cells, we observed a distinct morphological change in the cells overexpressing DAL-1. The A549 cells expressing DAL-1 underwent a phenotypic change and appear planar epithelial morphological shape, while the control A549 cells (Vector/A549) appear fibroblast-like mesenchymal cell morphological shape (Figure 2A).

Real-time quantitative PCR was used to assess the mRNA levels of EMT markers. The details of primer sequences for target genes were showed in Additional file 2: Table S1. As shown in Figure 2B,E-cadherin and β-catenin mRNAs were increased while Vimentin and N-cadherin mRNAs were decreased in DAL-1 stable transfected cells (*P <0.05, #*P* <0.01). Western blots also confirmed that DAL-1 overexpression resulted in an increment in E-cadherin and β-catenin expression and a reduction in Vimentin and N-cadherin expression at the protein level (Figure 2C).

**Figure 3:**
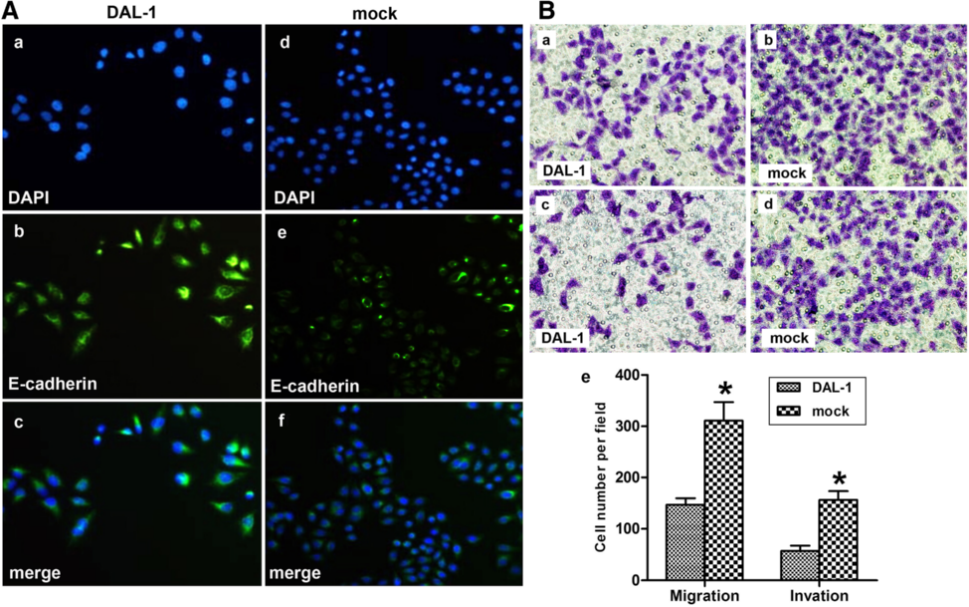
**DAL-1 attenuated cell migration and invasion in A549 cells. A**, mock and DAL-1 overexpressed A549 cells were stained as described under “Experimental Procedures” and examined using confocal immunofluorescence microscopy, as follows: DAPI (blue, top panel) and E-cadherin (green). Merged images are in the bottom of each panel. **B**, **a-b**, Cell migration assay was performed after reintroduction of DAL-1 in A549 cells. **c-d**, Matrigel cell invasion assay was performed after re-expression of DAL-1 in A549 cells. **e**, The data are presented as mean ± SD for triplicate determinations, *P <0.05.

We utilized the E-cadherin antibody and confocal microscopy to image E-cadherin expression in control and in DAL-1-overexpressed A549 cells. Figure 3A demonstrated that control A549 cells express relatively low levels of E-cadherin, whereas DAL-1-overexpressed cells expressed a great deal of E-cadherin localized in the plasma membrane.

In our previous studies, we found that DAL-1 affected cell proliferation in lung cancer cells. Herein, the role of DAL-1 in cell migration and invasion was evaluated by the Matrigel-based transwell invasion assay. The images showed that DAL-1 overexpression inhibited cell migration (Figure 3B, a-b) and invasion (Figure 3B, c-d) of A549 cells. The result of statistical analysis was showed in Figure 3B, e (*P <0.05).

### DAL-1 deficiency alters the expression of EMT markers

**Figure 4:**
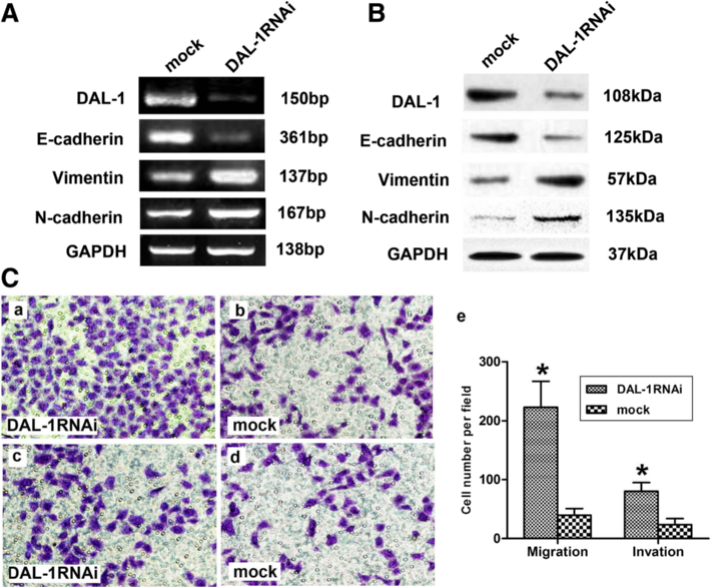
**DAL-1 deficiency alters the expression of EMT markers and metastasis ability. A**, RNA extracts of control and DAL-1 knockdown NCI-H460 cells were subjected to RT-PCR to determine the expression of target genes as indicated. **B**, The expression of EMT markers E-cadherin, Vimentin and N-cadherin were measured in control and DAL-1 knockdown NCI-H460 cells by western blotting. **C**, Cell migration assay **(a-b)** and invasion assay **(c-d)** were performed after knockdown of DAL-1 in NCI-H460 cells. **e**, The data are presented as mean ± SD for triplicate determinations, *P <0.05.

In the present study, we have observed an apparent endogenous expression of DAL-1 in NCI-H460 cells. To address the role of DAL-1 in NCI-H460 non-small cell lung cancer cell line, control and DAL-1 deficient cells were developed using non-silencing shRNA as a control and shRNA targeting DAL-1. As expected, DAL-1 knockdown resulted in reduction of E-cadherin mRNA expression level whereas Vimentin and N-cadherin mRNA levels were increased (Figure 4A). Similar results were obtained at the protein levels utilizing western blotting (Figure 4B). The Matrigel-based transwell invasion assay showed that DAL-1 knockdown promoted cell migration (Figure 4C, a-b) and invasion (Figure 4C, c-d) of A549 cells. Collectively, these data suggest that DAL-1 could inhibit EMT in lung cancer cell lines. The result of statistical analysis was showed in Figure 4C, e (*P <0.05).

### DAL-1 transcriptionally regulates E-cadherin

**Figure 5:**
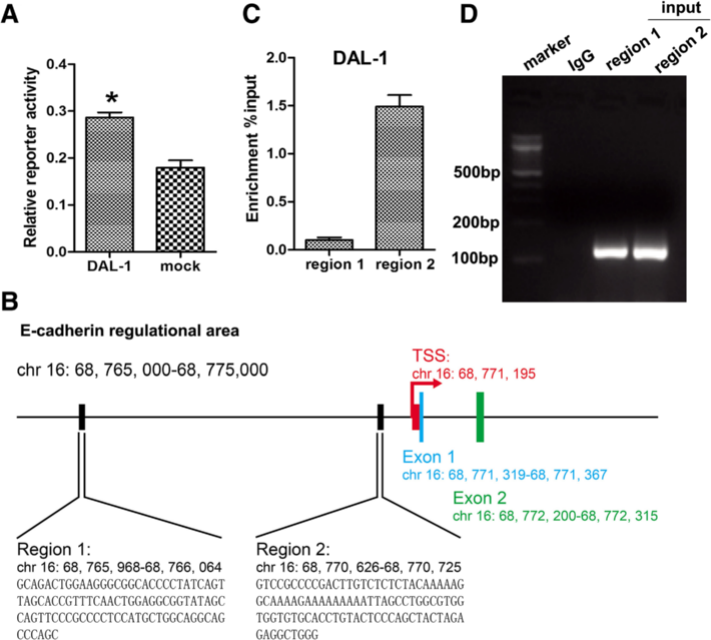
**DAL-1 transcriptionally regulates E-cadherin. A**, Dual-luciferase reporter assay showed that DAL-1 regulated E-caderin at the transcriptional level. The data are presented as the mean ± SD. n = 6, *P <0.05. **B**, Schematic representation of the E-cadherin regulational area. Two regions were chosen to design the primers for qPCR. **C**, The result DNA from chromatin immunoprecipitation assay was analyzed by qPCR. **D**, Different primers were compounded and analyzed by PCR.

Although it had been confirmed that expression of DAL-1 had positive correlation with E-cadheirn, we asked whether DAL-1 could regulate the activity of the E-cadherin promoter using a dual-luciferase reporter assay. Empty pcDNA3 vector or pcDNA3-DAL-1 plasmids were transfected into A549 cells together with an E-cadherin promoter. We examined the effect of DAL-1 overexpression or depletion on E-cadherin promoter luciferase reporter activity in the region of upstream 450 bp to downstream 193 bp. Figure 5A demonstrated that E-cadherin promoter activity was increased in DAL-1 overexpressed A549 cells comparing to the control cells (*P <0.05). These finding suggest that DAL-1 may impact on EMT through directly transcriptionally regulating E-cadherin.

To directly confirm the binding of E-cadherin promoter to the endogenous DAL-1 protein in vivo, we performed ChIP assays in NCI-H460 cells by using anti-DAL-1 antibody. Figure 5B showed the schematic representation of the E-cadherin regulational area. Two regions were chosen to design the primers for qPCR. The result of qPCR analysis demonstrated that E-cadherin promoter (region 2) bound DAL-1 in vivo (Figure 5C). Different primers were compounded and analyzed by PCR. The PCR products were found using primers set spanning E-cadherin but none with IgG control primers (Figure 5D). The results showed that DAL-1 bound to the region 2 of E-cadherin promoter in vivo.

### HSPA5 as a direct associated protein to DAL-1

**Figure 6:**
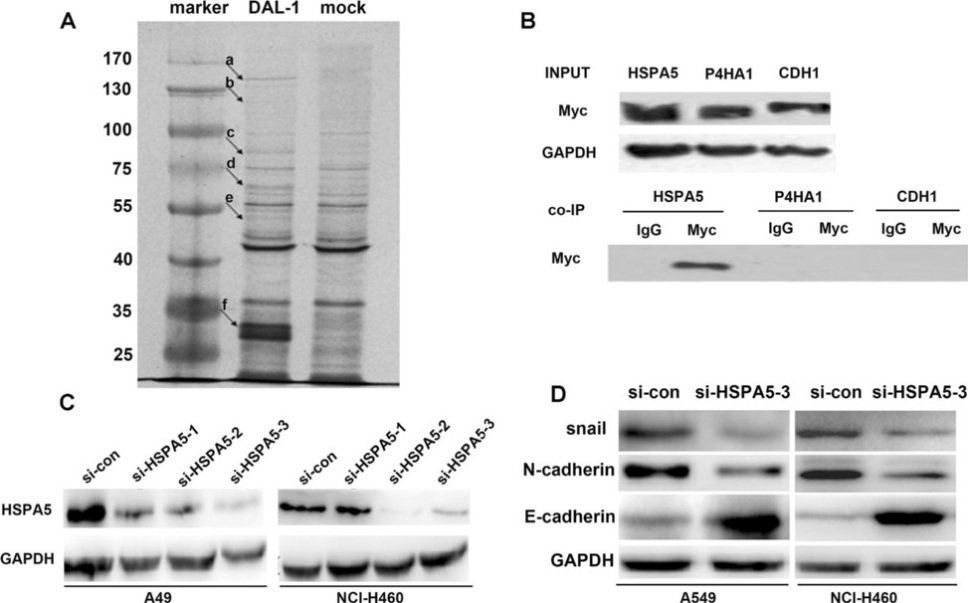
**HSPA5 is the direct binding protein to DAL-1 which contributes positively to EMT in lung cancer. A**, Co-immunoprecipitation and mass spectrometric assay were performed to show the binding proteins to DAL-1. **a**, 4.1B; **b**, E-cadherin; **c,** HSPA5; **d**, P4HA1; **e**, Tubulin beta chain; **f**, 14-3-3ε. **B**, Endogenous co-immunoprecipitation analysis conformed that HSPA5 was the direct binding protein to DAL-1. **C**, The function of three designed HSPA5 RNA interference was determined by western blotting in A549 and NCI-H460 cells. **D**, The expression of EMT markers E-cadherin, N-cadherin and snail were measured in control and HSPA5 knockdown A549 or NCI-H460 cells by western blotting.

Previous investigation highlighted that DAL-1 located in the lateral membrane of various epithelial cells, suggesting it could participate in adherent junction. We performed co-immunoprecipitation and mass spectrometric assay to examine the binding proteins to DAL-1. Figure 6A showed that the protein complex was co-immunoprecipitated with the DAL-1 antibody-coupled resin and followed SDS-PAGE for separation. Sliver staining image showed that six differentially bands appeared in the DAL-1 expression group while no specific band was observed at the same position of the control group. The gel of the bands was sent to MALDI TOF/TOF Mass Spectrometry for further analysis. Protein identification was finished by searching the PMF (Peptide Mass Fingerprint). Mascot search results demonstrated the details of masss pectrometric data (Additional files 3, 4, 5, 6, 7 and 8). The results from Additional file 2: Table S2 showed five differentially proteins including E-cadherin, HSPA5, P4HA1, Tubulin beta chain and 14-3-3ε.

Further we investigated the association proteins by using co-immunoprecipitation experiment, whether they interacted with each other in lung cancer cells were tested. As shown in Figure 6B, HSPA5 was co-immunoprecipitated with DAL-1, whereas HSPA5 was not detected when applied with control IgG. The results indicated that DAL-1 associated with HSPA5 directly. Previous investigations have shown that HSPA5 may be an oncogene existing in cells. Moreover, we verified that HSPA5 konckdown could interfere with EMT by using RNA interference (RNAi) (Figure 6C). HSPA5 knockdown resulted in reduction of snail and N-cadherin expression levels whereas E-cadherin proein level was increased (Figure 6D). Evidence of HSPA5 promoted the EMT process together with previous results, it would be important to note that DAL-1 might inhibit HSPA5 then impact on EMT in lung cancer. However, we have not yet identified the partner of DAL-1 involved in this process and the mechanism linked to HSPA5 remains to be investigated.

## Discussion

Tumor metastasis of is a complex multi-step and multi-stage process [13,14]. The mortality of lung cancer is often related to extensive metastasis [15,16]. During the metastasis cascade, carcinoma cells often activate EMT to underlie metastasis by promoting acquisition of migratory and invasive abilities [17-19]. Therefore, understanding the mechanisms inducing EMT is particularly important for developing strategies for clinical therapy [20-22]. DAL-1 was originally considered as a tumor growth suppressor gene based on its downregulation in lung adenocarcinoma [23,24]. Recent research reported that DAL-1 could suppress tumor metastasis [25].

As DAL-1 is associated with the cytoskeleton molecules and can regulate the cellular functions in adhesion and migration under physiological conditions [26,27], we speculated that DAL-1 might suppress metastasis via inhibiting EMT. Forced DAL-1 expression, however, abolished the metastatic capacity of a fibroblastoid, highly metastatic human lung cancer cell line through reversal to an epithelial phenotype. Loss of DAL-1 also predicted metastasis and impaired survival in a lung cancer tissue arrays from 190 patients, suggesting DAL-1 as an excellent biomarker for human metastatic lung cancer. Since DAL-1 abolishes metastatic capacity by re-inducing epithelial properties in dedifferentiated tumour cells, it might also be a candidate for gene therapy in metastatic cancer. DAL-1 overexpression altered the EMT markers expression, the expression of E-cadherin and β-catenin were upregulated, and expression of vimentin and N-cadherin were downregulated. Furthermore, inhibition of DAL-1 dramatically promoted the migration and invasion abilities of lung cancer cells. Our data together suggest that DAL-1 might contribute to the development of EMT.

Studies to unravel possible mechanisms, how loss of DAL-1 disturbs epithelial polarity with the outcome of EMT, are hindered by the enormous complexity of these processes. E-cadherin was observed to be upregulated after DAL-1 reexpression. Consistent with previous report, we confirmed that E-cadherin upregulation was sufficient to inhibit EMT in lung cancer cells [28]. We demonstrated that DAL-1 might increase the expression of E-cadherin via transcriptional activating the E-cadherin promoter. Our result of the dual-luciferase reporter assay and ChIP analysis identified that DAL-1 could bind E-cadherin promoter directly and increase expression of E-cadherin as a transcriptional factor. Recent study identified a putative NSL (nuclear localization signal) in exon 12 of DAL-1 [29]. Interestingly, a casein kinase II site, SAAE, is 26 amino acids upstream of the NLS in exon 12. It has been reported that proteins containing an NLS often have casein kinase II site 10–30 amino acids from the NLS [30]. The conformation of DAL-1 would be changed if the kinase site was phosphorylated, and the NLS would be exposed to be identified, leading DAL-1 into nuclear to act a role as transcriptional factor.

DAL-1 as an important component of membrane-associated cytoskeleton was originally detected in cell-cell junction part. DAL-1 contains some specific domains, including SABD and FERM domains [31,32] which lead DAL-1 function as a scaffold protein binding to the cell membrane and connecting to the cytoskeleton [27,33,34]. This kind of structure is essential for the maintainance of cell polarity and cell-cell adhesion [35,36]. Loss of DAL-1 may cause impairing of this structure and result in EMT development.

DAL-1 associated proteins were investigated by co-immunoprecipitation assay. E-cadherin, β-tubulin, P4HA1, 14-3-3ε and HSPA5 had been found in the DAL-1 binding complex. Previous research showed that DAL-1 interacted with 14-3-3ε and contributed to cell apoptosis [37,38]. So we performed endogenous co-immunoprecipitation experiment to conform the direct interaction between DAL-1 and P4HA1, E-cadherin or HSPA5. P4HA1 as a Prolyl hydroxylase family member could upregulate E-cadherin expression via induce HIF degradation [39,40]. The results showed that only HSPA5 was the combined protein to DAL-1. Molecular chaperone HSPA5 was a key survival factor in development and cancer [41]. HSPA5 may also be important for tumor metastasis because it is elevated in metastatic cancer cell lines, lymph node metastasis, and knockdown of HSPA5 inhibits tumor cell invasion in vitro and growth and metastasis in xenograft models [42,43]. The notable component of our work described here was that HSPA5 interacted with DAL-1 directly. HSPA5 knockdown could impact on EMT markers. Whether DAL-1 suppresses EMT via inhibiting HSPA5, further investigations will explore this mechanism.

## Conclusions

In this study, we have demonstrated that the endogenous expression of DAL-1 was negatively correlated with the tumor pathological grading of clinical lung cancer samples. Further in vitro experiments have showed that DAL-1 reduced migration and invasion, transcriptional activated E-cadherin promoter. Moreover, HSPA5 as a DAL-1 co-precipitated protein contributed in EMT process. Based on our observations and results reported by other groups, we have proposed DAL-1 could attenuate EMT and be important for tumor metastasis in the early transformation process of lung cancer. The experimental data and conclusions in the present study furnish valuable information regarding the biological functions of DAL-1 and the possible mechanism of the migration and invasion of lung tumor. Thus, further studies will be focused on the molecular network involved in the DAL-1 regulation on EMT.

## Additional files

Additional file 1:
**Expression of DAL-1 in lung cancer cell lines.** The DAL-1 mRNA expression levels (A) and protein expression levels (B) were detected by RT-PCR and western blotting in randomly selected seven lung cancer cell lines A549, SPC-A1, PGC-L3, GLC-82, H1299, NCI-H460 and L78. (G: GAPDH, 145bp, D: DAL-1, 154bp). Additional file 2: Table S1. Primers used in Quantitative Real-time PCR. **Table S2.** Immunopricipitated proteins were identified by MALDI TOF/TOF Mass Spectrometry. Additional file 3:
**Mascot search result of 4.1B demonstrated by mass spectrometric analysis.**
Additional file 4:
**Mascot search result of E-cadherin demonstrated by mass spectrometric analysis.**
Additional file 5:
**Mascot search result of HSPA5 demonstrated by mass spectrometric analysis.**
Additional file 6:
**Mascot search result of P4HA1 demonstrated by mass spectrometric analysis.**
Additional file 7:
**Mascot search result of Tubulin beta chain demonstrated by mass spectrometric analysis.**
Additional file 8:
**Mascot search result of 14-3-3ε demonstrated by mass spectrometric analysis.**

## Abbreviations

DAL-1: Differentially expressed in adenocarcinoma of the lung-1
EMT: Epithelial-to mesenchymal transition
NSCLC: Non small cell carcinoma
HSPA5: Heat shock protein A5
