# Supplementary figures and images for: DAL-1 attenuates epithelial-to mesenchymal transition in lung cancer
Source: J Exp Clin Cancer Res. 2015 Jan 22;34(1):3. doi: 10.1186/s13046-014-0117-2 (PMC4307741; doi:10.1186/s13046-014-0117-2)

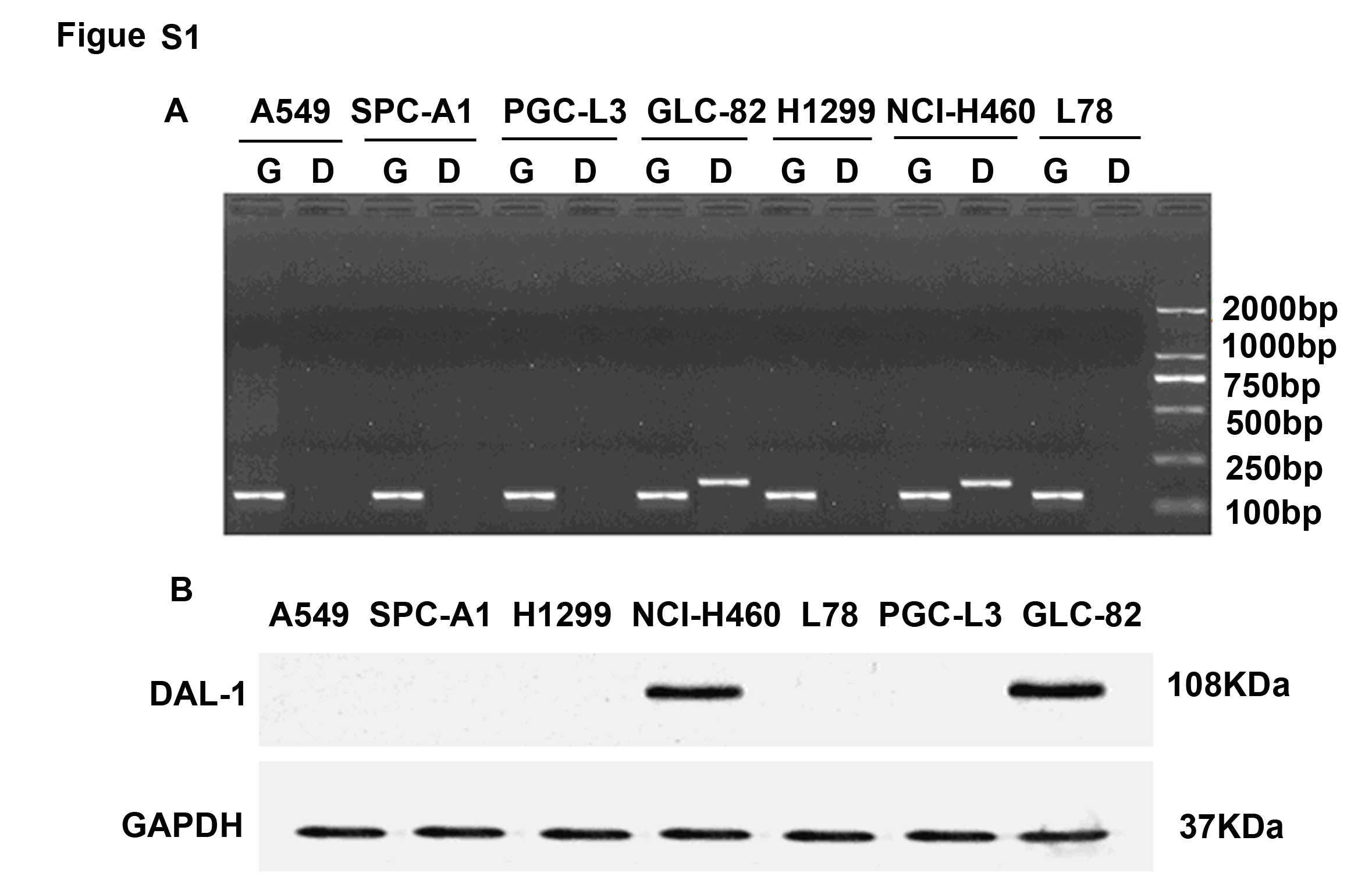

Supplement: Additional file 1: — Expression of DAL-1 in lung cancer cell lines. The DAL-1 mRNA expression levels (A) and protein expression levels (B) were detected by RT-PCR and western blotting in randomly selected seven lung cancer cell lines A549, SPC-A1, PGC-L3, GLC-82, H1299, NCI-H460 and L78. (G: GAPDH, 145bp, D: DAL-1, 154bp). [file 13046_2014_117_MOESM1_ESM.jpeg]

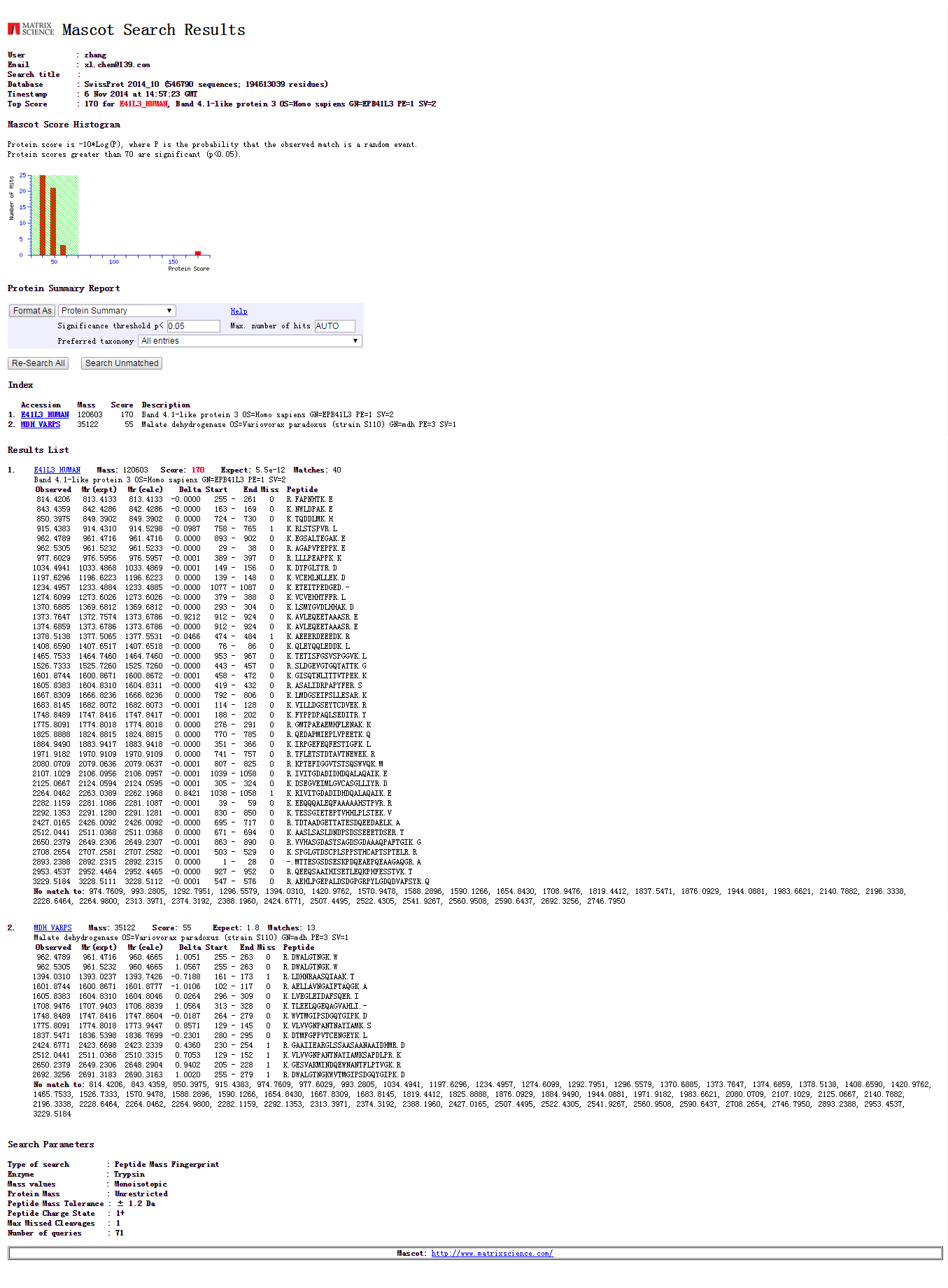

Supplement: Additional file 3: — Mascot search result of 4.1B demonstrated by mass spectrometric analysis. [file 13046_2014_117_MOESM3_ESM.jpeg]

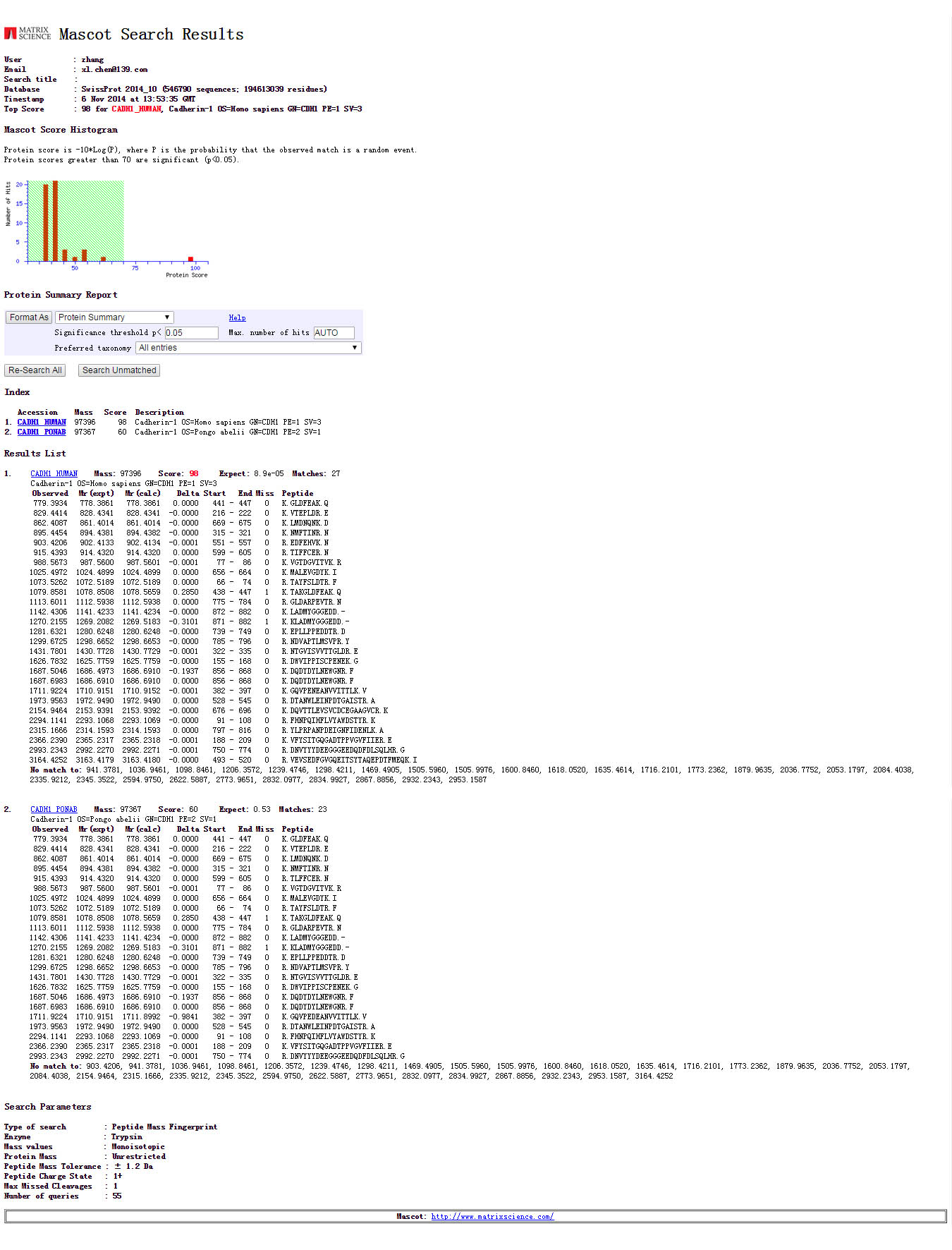

Supplement: Additional file 4: — Mascot search result of E-cadherin demonstrated by mass spectrometric analysis. [file 13046_2014_117_MOESM4_ESM.jpeg]

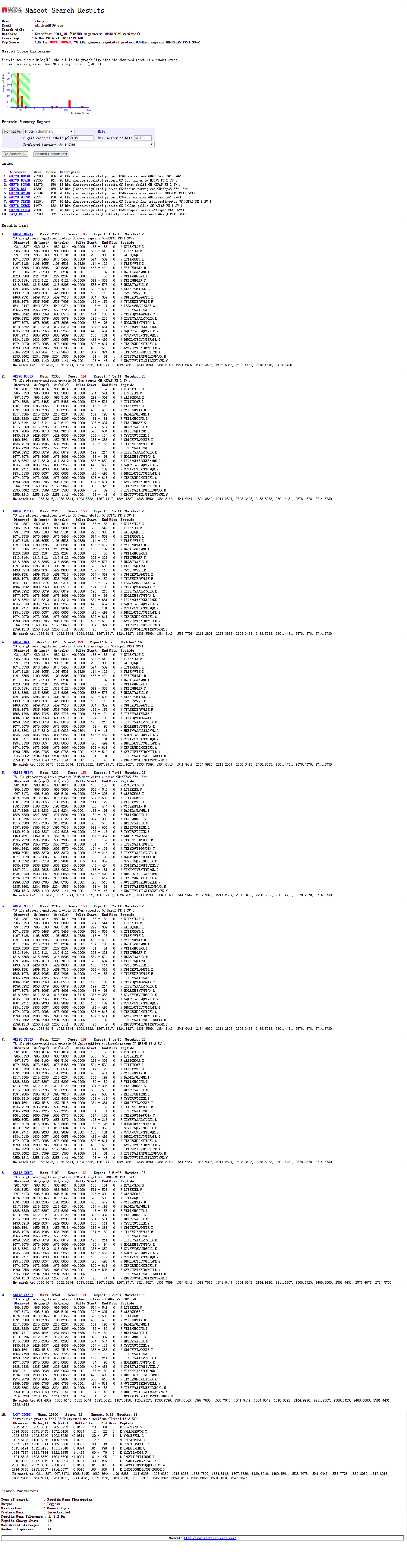

Supplement: Additional file 5: — Mascot search result of HSPA5 demonstrated by mass spectrometric analysis. [file 13046_2014_117_MOESM5_ESM.jpeg]

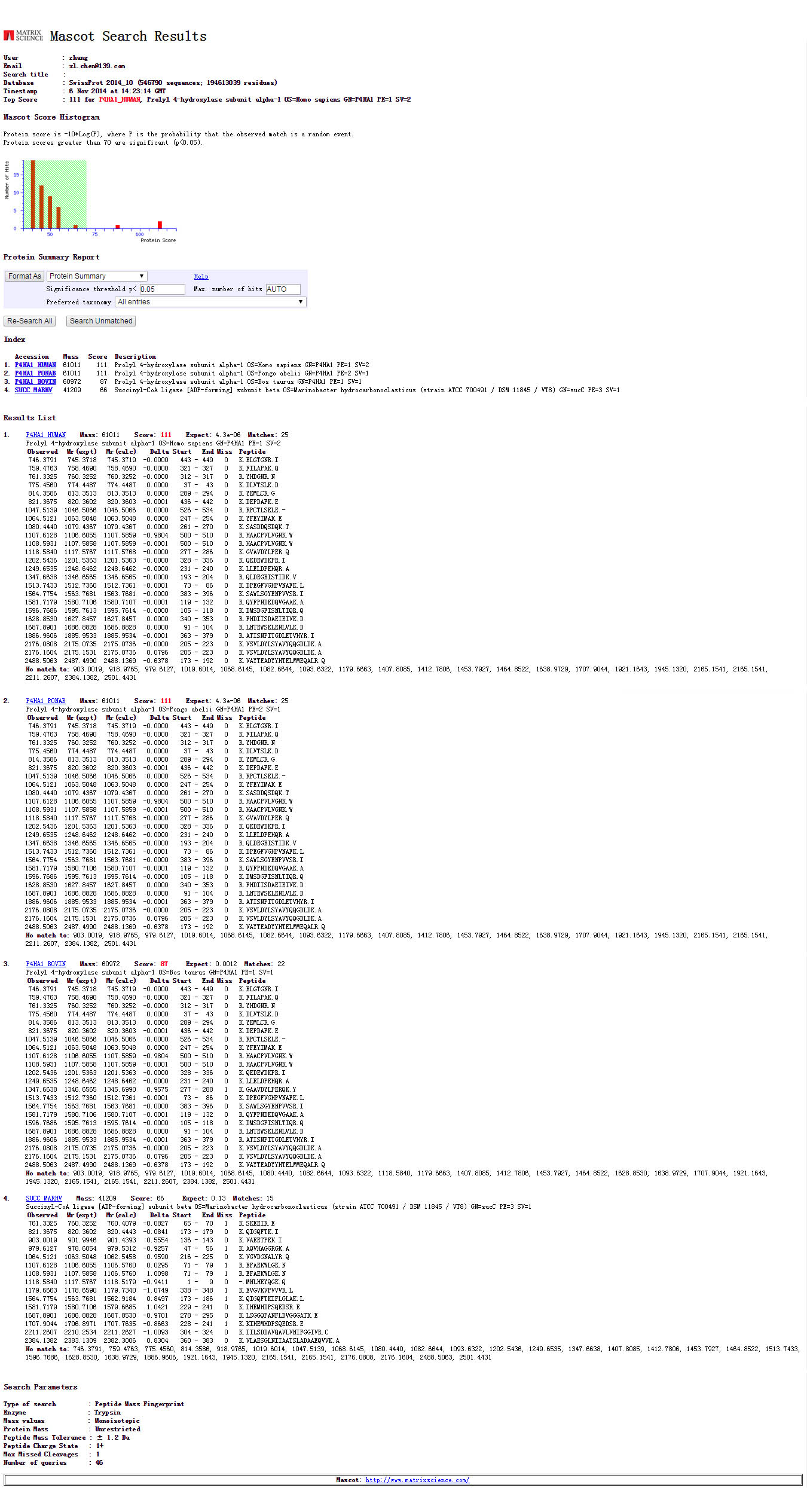

Supplement: Additional file 6: — Mascot search result of P4HA1 demonstrated by mass spectrometric analysis. [file 13046_2014_117_MOESM6_ESM.jpeg]

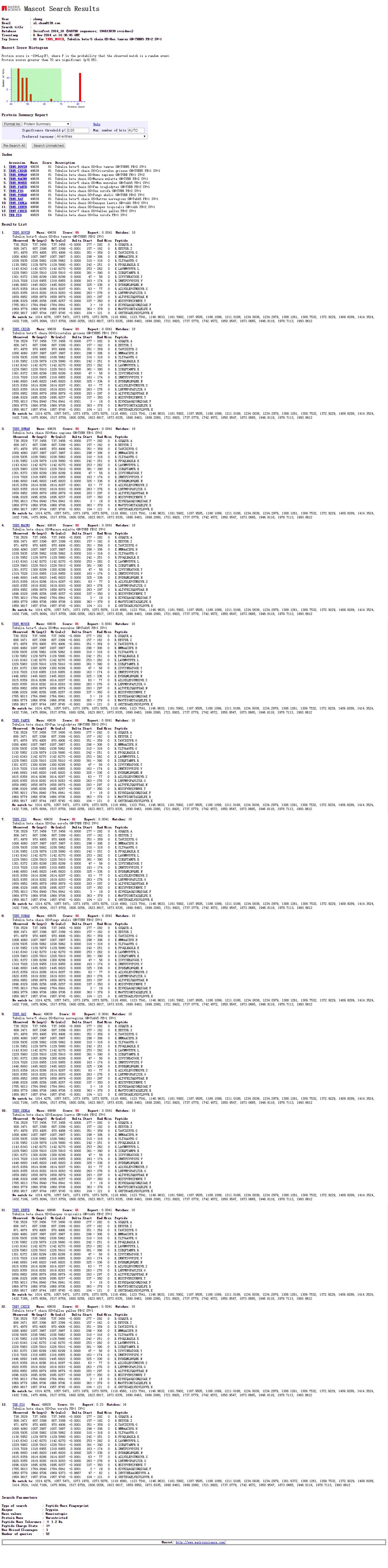

Supplement: Additional file 7: — Mascot search result of Tubulin beta chain demonstrated by mass spectrometric analysis. [file 13046_2014_117_MOESM7_ESM.jpeg]

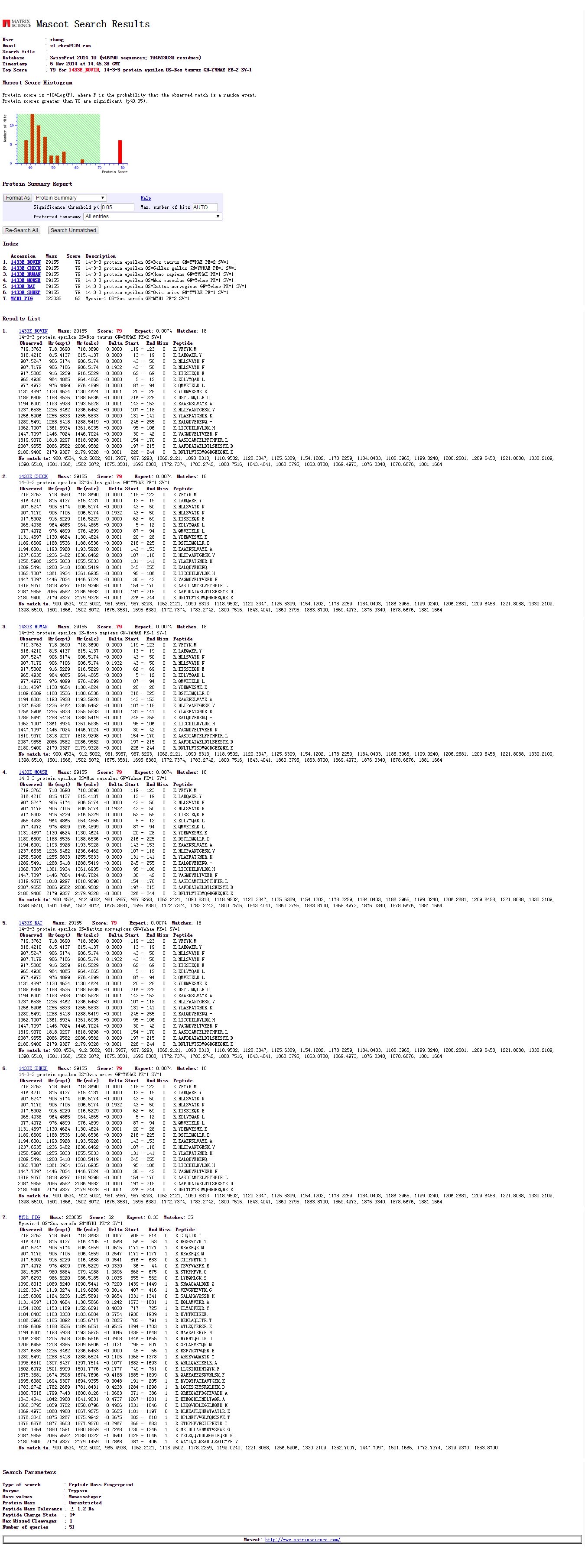

Supplement: Additional file 8: — Mascot search result of 14-3-3ε demonstrated by mass spectrometric analysis. [file 13046_2014_117_MOESM8_ESM.jpeg]
