# Supplementary material for: DAL-1 attenuates epithelial-to mesenchymal transition in lung cancer
Source: J Exp Clin Cancer Res. 2015 Jan 22;34(1):3. doi: 10.1186/s13046-014-0117-2 (PMC4307741; doi:10.1186/s13046-014-0117-2)
Supplement: Additional file 2: Table S1. — Primers used in Quantitative Real-time PCR. Table S2. Immunopricipitated proteins were identified by MALDI TOF/TOF Mass Spectrometry. [file 13046_2014_117_MOESM2_ESM.doc]

**Supporting Information**

**Material and Methods**

**Reverse transcription and real-time quantitative PCR**

Total RNA was extracted using Trizol Reagent (Invitrogen, Carlsbad, CA, USA). RT was performed using 1 μg total RNA and the PrimeScript RT kit (Takara Bio Inc, Japan). PCR was carried out in 25 μl reactions using GoTaq Green Master Mix (Promega, Madison, WI, USA) in a MJ Research PTC-200 Thermal Cycler system (MJ Research Inc, Waltham, MA, USA). PCR products were resolved by 1 % agarose gel electrophoresis, stained with SYBR green, and detected using a Kodak Image Station 4000R (Carestream Health Inc, Rochester, NY, USA).

Real-time PCR was performed using the SYBR®Premix Ex Taq™ II (Perfect Real Time) reagent (TAKARA) following the manufacturer’s instructions. The PCR reaction of 20 μl contained 200 nM of primers and 20 ng of cDNA template. The amplification was monitored using an ABI PRISM 7000 Sequence Detection System (Applied Biosystems Inc, Carlsbad, CA, USA). Real-time qPCR detection threshold cycle (CT) values were generated with the system software. The relative concentration of each transcript was calculated using the Pfaffl method. Efficiency for each gene was determined from five-point serial dilutions of positive control cDNA samples. PCR was executed according to the manufacturer's instructions. The cycling conditions included a holding step at 94 ˚C for 3 min and 29 cycles of 94 ˚C for 30 s; 55 ˚C for 30 s and 72 ˚C for 1 min. Relative quantification were analyzed using the ΔΔCT method. All experiments were repeated three times. The details of primer sequences for target genes were showed in **Table S1**

**Protein isolation and measurement by western blot**

Cells were homogenized in lysis buffer containing 250 mM cane sugar, 100 mM NaCl, 50 mM KCl, 20 mM Tris-Cl, and 1 mM PMSF. The total protein concentration was determined by a Protein Analyzer (Pharmacia Biotech, Piscataway, NJ, USA). Equal amounts of total protein for each sample were loaded on an sodium dodecyl sulfate-polyacrylamide gel electrophoresis gel and blotted with polyclonal antibodies specific for DAL-1 (Santa Cruz Biotechnology, Santa Cruz, CA, USA), monoclonal antibody against GAPDH, E-cadherin, β-catenin, Vimentin, Fibronectin and Snail (Cell Signaling, Danvers, MA, USA) in 5 % non-fat dry milk in Tris-buffered saline containing 0.1 % Tween® 20. After incubating with the corresponding second antibody, the results were visualized using the LumiGLO™ Chemiluminescent Substrate solution (Cell Signaling, Danvers, MA, USA).

**Results**

**DAL-1 is down-regulated in cell lines**

Several kinds of lung cancer cell lines, including A549, SPC-A1, PGC-L3, GLC-82, H1299, L78, and NCI-H460 were analyzed the DAL-1 mRNA and protein expression levels. GLC-82 and NCI-H460 cell lines were positive for DAL-1 mRNA and protein expression as indicated by RT-PCR products at the expected size of 153bp and the specific protein binding band at the predicted size of 108 kDa (Additional file 2). The endogenous DAL-1 expression had not been found in other kinds of cell lines.

**Figure legends**

**Additional file 2.** **Expression of DAL-1 in lung cancer cell lines**. The DAL-1 mRNA expression levels (**A**) and protein expression levels (**B**) were detected by RT-PCR and western blotting in randomly selected seven lung cancer cell lines A549, SPC-A1, PGC-L3, GLC-82, H1299, NCI-H460 and L78. (G: GAPDH, 145bp, D: DAL-1, 154bp)

**Additional file 3. Mascot search result of 4.1B demonstrated by mass spectrometric analysis.**

**Additional file 4. Mascot search result of E-cadherin demonstrated by mass spectrometric analysis.**

**Additional file 5. Mascot search result of HSPA5 demonstrated by mass spectrometric analysis.**

**Additional file 6. Mascot search result of P4HA1 demonstrated by mass spectrometric analysis.**

**Additional file 7. Mascot search result of Tubulin beta chain demonstrated by mass spectrometric analysis.**

**Additional file 8. Mascot search result of 14-3-3ε demonstrated by mass spectrometric analysis.**

Table S1. Primers used in Quantitative Real-time PCR.

| **Target gene** | **Forward primer** | **Reverse primer** |
| --- | --- | --- |
| E-cadherin | 5’- caatgccgccatcgcttac-3’ | 5’- atgactcctgtgttcctgttaatg -3’ |
| Vimentin | 5’-gagaactttgccgttgaagc-3’ | 5’-tccagcagcttcctgtaggt-3’ |
| Snail | 5’-gctccacaagcaccaagagt-3’ | 5’-attccatggcagtgagaagg-3’ |
| Fibronectin | 5’-accaacctacggatgactcg-3’ | 5’-gctcatcatctggccatttt-3’ |
| β-catenin | 5’-gtacgtccatgggtgggaca-3’ | 5’- ggctccggtacaaccttcaacta-3’ |
| DAL-1 | 5’-gagctgccaagcgtttatgga-3’ | 5’-cctgccactataacgaaacttggaa-3’ |
| N-cadherin | 5’- attggaccatcactcggctta-3 | 5’-cacactggcaaaccttcacg-3’ |
| GAPDH | 5’- gcaccgtcaaggctgagaac -3’ | 5’- tggtgaagacgccagtgga -3’ |

**Table S2. Immunopricipitated proteins were identified by MALDI TOF/TOF Mass Spectrometry.**

| **No.** | **IPI number** | **Protein name** | **MW** | **Protein Score** | **Protein Score C.I.%** |
| --- | --- | --- | --- | --- | --- |
| a | 00032230 | 4.1B | 120603.1 | 192 | 100 |
| b | 00744889 | **E-cadherin** | 99694 | 97 | 100 |
| c | 00003362 | **HSPA5** | 72288.4 | 192 | 100 |
| d | 00009923 | **P4HA1** | 61011 | 107 | 100 |
| e | 01019113 | Tubulin beta chain | 49639 | 86 | 99.995 |
| f | 00000816 | 14-3-3ε | 29155.4 | 80 | 100 |
